# Timing and Safety of Anticoagulation Reinitiation After Intracranial Hemorrhage in Patients With Mechanical Valves: A Meta-Analysis

**DOI:** 10.1212/WNL.0000000000214184

**Published:** 2025-09-25

**Authors:** Lucio D'Anna, Gabriele Prandin, Edoardo Pirera, Simona Sacco, Roland Veltkamp, Eleni Korompoki, Marta Gigli, Maurizio Paciaroni, Diana A. Gorog, Francesco Favruzzo, Prapa Kanagaratnam, Boom Lim, Liqun Zhang, Robert J. Simister, Mariarosaria Valente, Gian Luigi Gigli, Matteo Foschi, Giovanni Merlino

**Affiliations:** 1 Department of Stroke and Neuroscience, Charing Cross Hospital, Imperial College London NHS Healthcare Trust, United Kingdom;; 2 Department of Brain Sciences, Imperial College London, United Kingdom;; 3 Clinical Unit of Neurology, Department of Medicine, Surgery and Health Sciences, University Hospital and Health Services of Trieste, ASUGI, University of Trieste, Italy;; 4 Internal Medicine and Stroke Care Ward, Department of Promoting Health, Maternal-Infant, Excellence and Internal and Specialized Medicine (ProMISE) “G. D'Alessandro,” University of Palermo, Italy;; 5 Department of Biotechnological and Applied Clinical Sciences, University of L'Aquila, Italy;; 6 Department of Neurology, Alfried-Krupp Krankenhaus Essen, Germany;; 7 Department of Neurology, University Hospital Heidelberg, Germany;; 8 Department of Clinical Therapeutics, National and Kapodistrian University of Athens, Greece;; 9 Cardiovascular Department, Azienda Sanitaria-Universitaria Giuliano Isontina (ASUGI), Trieste, Italy;; 10 Department of Neurosciences and Rehabilitation, University of Ferrara, Italy;; 11 Faculty of Medicine, National Heart and Lung Institute, Imperial College, London, United Kingdom;; 12 School of Life and Medical Sciences, Postgraduate Medical School, University of Hertfordshire, United Kingdom;; 13 Comprehensive Stroke Center, Ospedale dell'Angelo, Azienda ULSS 3 Serenissima, Mestre, Italy;; 14 Department of Cardiology, Hammersmith Hospital, Imperial College London NHS Healthcare Trust, United Kingdom;; 15 Stroke Unit, Department of Neuroscience, George's University of London, United Kingdom;; 16 Comprehensive Stroke Service, University College London Hospital, United Kingdom;; 17 Clinical Neurology, Udine University Hospital and Department of Medicine (DMED), University of Udine, Italy; and; 18 SOSD Stroke Unit, Department Head, Neck, and Neurosciences, Udine University Hospital, Italy.

## Abstract

**Background and Objectives:**

In patients with mechanical heart valves (MHVs), anticoagulation (AC) interruption after intracranial hemorrhage (ICH) poses a clinical dilemma because of competing risks of ischemic complications and hemorrhagic recurrence. To date, the optimal timing for resuming vitamin K antagonists (VKAs) remains unclear. The aim of this meta-analysis was to quantify the risks of ischemic stroke and recurrent ICH associated with VKA resumption in this population and explore the temporal risk dynamics.

**Methods:**

We systematically searched PubMed, Embase, and Cochrane Library from inception to December 2023 for studies reporting ischemic or hemorrhagic outcomes in adults with MHVs who experienced ICH and were considered for VKA resumption. Primary outcomes were ischemic stroke before AC resumption and recurrent ICH after AC resumption. Random-effects meta-analyses were performed. Meta-regressions assessed whether timing of resumption influenced risk. Risk trajectories were estimated using a model-based approach.

**Results:**

Nine studies were included, comprising 435 patients with MHVs with confirmed ICH included in the pooled analysis. The mean age ranged from 54.1 to 75 years; 31.3% were female. The pooled incidence of recurrent ICH after AC reinitiation was 11.4% (95% CI 8.2–15.6; *I*^2^ = 0%), the incidence of ischemic stroke during AC suspension was 6.1% (95% CI 4.1–8.9; *I*^2^ = 0%), valve thrombosis occurred in 3.3% (95% CI 1.9–5.6; *I*^2^ = 0%), and mortality occurred in 4.9% (95% CI 2.0–11.5; *I*^2^ = 37%). Meta-regression demonstrated a significant inverse association between time to AC resumption and risk of recurrent ICH (regression coefficient −0.039; 95% CI −0.093 to 0.015; *p* = 0.13), corresponding to an approximate 50% relative reduction in risk at 11 days after ICH. No significant time-dependent association was observed for ischemic stroke (coefficient −0.013; 95% CI −0.065 to 0.039; *p* = 0.61).

**Discussion:**

In patients with MHVs who experienced an ICH, this meta-analysis found that resumption of AC was associated with a recurrent ICH rate of 11.4% and an ischemic stroke rate of 6.1% during AC suspension. Meta-regression suggested a lower risk of recurrent ICH with later AC resumption, with a potential risk reduction at approximately 11 days after ICH. No time-dependent increase in ischemic stroke was observed. Limitations include the retrospective design of most studies and heterogeneous AC timing across cohorts.

## Introduction

Anticoagulation (AC) with vitamin K antagonists (VKAs) is essential for preventing thromboembolic complications in patients with mechanical heart valves (MHVs).^1-3^ However, managing these patients becomes particularly challenging when AC must be interrupted because of intracranial hemorrhage (ICH), one of the most feared and potentially fatal complications of anticoagulant therapy.^4-6^ Clinical decision making in this context involves balancing 2 competing risks: the risk of thromboembolic events such as acute ischemic stroke (AIS) or valve thrombosis when AC is withheld, and the risk of ICH recurrence when AC is resumed.^7^ Current international guidelines provide limited and often vague recommendations regarding the optimal timing for resumption of AC in patients with MHVs after ICH.^8^ This is largely due to the lack of high-quality randomized trials in this population and the rarity of patients with mechanical valves among broader anticoagulated cohorts. Consequently, clinical practice varies widely, and decisions are frequently made on a case-by-case basis. We, therefore, conducted a systematic review and meta-analysis to estimate the pooled risk of ischemic stroke, valve thrombosis, recurrent ICH, and death in patients with MHVs who resume AC after ICH. In addition, we explored whether the timing of AC reinitiation modifies the risk of adverse events, with the goal of identifying time thresholds that may guide clinical practice.

## Methods

### Study Design

This systematic review and meta-analysis was registered on the International Prospective Register of Systematic Reviews (PROSPERO, CRD CRD420251022500) and reported in accordance with the Preferred Reporting Items for Systematic Review and Meta-Analysis (PRISMA) statement guidelines. The primary objective of this systematic review and meta-analysis was to assess the key outcomes after VKA reinitiation following ICH in patients with MHVs.

### Standard Protocol Approvals, Registrations, and Patient Consents

This systematic review and meta-analysis was conducted in accordance with ethical standards for research involving human participants. Ethical approval for the conduct of this study was not required because all data were obtained from previously published studies. For all included studies, approval by institutional or regional ethics committees was presumed based on original publications. Because this analysis did not involve direct patient contact or identifiable individual-level data, patient consent was not required. No images or other identifiable materials requiring disclosure consent were included.

### Data Source and Search Strategy

We systematically searched for peer-reviewed reports without language and time restrictions in PubMed, Embase, Web Of Science, Cochrane Central Register of Controlled Trials (CENTRAL), and Cochrane Reviews from inception to 22 April 2025. The search term strategy included a combination of synonyms of “heart valve prosthesis,” “intracranial hemorrhage,” and “anticoagulant.” Both Medical Subject Headings terms and relevant free-text keywords were used. We further used a snowballing approach by manually screening the reference lists of the included studies and relevant previously published review articles. The detailed search strategy is provided in eTable 1.

### Eligibility Criteria

For the purpose of this systematic review and meta-analysis, we included studies that met all the following eligibility criteria: (1) enrolled patients aged 18 years or older with MHVs; (2) included patients admitted to hospital with spontaneous ICH as the main diagnosis; (3) randomized controlled trials, nonrandomized controlled trials, cohort studies, and case series; (4) reported the outcomes of interest; (5) included patients with MHVs alongside other indications for VKAs only when patients with MHVs comprised over half of those who reinitiated VKA treatment. We excluded studies involving pregnant patients.

### Quality Assessment

The risk of bias in nonrandomized studies was evaluated using the Risk Of Bias In Non-randomized Studies of Interventions (ROBINS-I) tool, which assesses 7 domains: confounding, selection of participants, classification of interventions, deviations from intended interventions, missing data, measurement of outcomes, and selection of the reported result.

### Outcomes and Data Extraction

The study outcomes of interest were (1) ischemic stroke before VKA reinitiation after ICH, (2) occurrence of valve thrombosis before VKA reinitiation after ICH, () recurrence of symptomatic ICH after VKA reinitiation following ICH, and (4) any death after VKA reinitiation following ICH. Data extraction was independently performed by 2 authors (L.D. and G.P.), and data were entered into an electronic spreadsheet. Disagreements in data extraction were resolved through discussion between the 2 authors. In cases of persistent disagreement, a third reviewer provided a final opinion to reach consensus (G.M.). The following baseline characteristics were collected from each study: study design and period, duration of follow-up, number of participants, median or mean time off AC, age, sex, and presence of atrial fibrillation.

### Statistical Analysis

We conducted single-arm meta-analyses using random-effects models to estimate pooled proportions for each outcome. Proportions were pooled using the logit transformation method with the inverse variance approach. Between-study heterogeneity was estimated using the DerSimonian-Laird method. Studies with zero events were handled by applying a continuity correction of 0.5 to all cells. Heterogeneity was assessed using the *I*^2^ statistic and Cochran *Q* test, with an *I*^2^ value of 0%–30% indicating low heterogeneity, 31%–60% moderate heterogeneity, 61%–90% substantial heterogeneity, and 91%–100% considerable heterogeneity. *p* Values <0.10 for the *Q* test were considered statistically significant for heterogeneity. A sensitivity analysis was conducted by sequentially removing 1 study at a time to assess the robustness of the pooled estimates and the influence of individual studies. This leave-one-out analysis was performed using the metainf function from the “meta” package in R. To investigate whether the timing of VKA reinitiation influenced the risk of ischemic stroke and ICH, we performed meta-regression analyses using the number of days off AC as a continuous moderator. When studies reported medians instead of means for timing, medians were used directly, and this was explicitly acknowledged as a methodological limitation due to potential data skewness. Based on the meta-regression coefficients, we generated time-dependent risk trajectories for both outcomes over a 30-day period. Specifically, we estimated the time points at which the risk of recurrent ICH would be reduced by 25%, 50%, and 75% compared with baseline and the risk of ischemic stroke would be increased by 25%, 50%, and 75%. These inflection points were used to illustrate the dynamic balance between thromboembolic and hemorrhagic risks over time and to help identify potential windows of optimal safety for AC resumption. Statistical analyses were performed using R version 4.4.0 (R Foundation for Statistical Computing, Vienna, Austria) with the “meta” package.

### Data Availability

Data are available on reasonable request.

## Results

**Figure 1:**
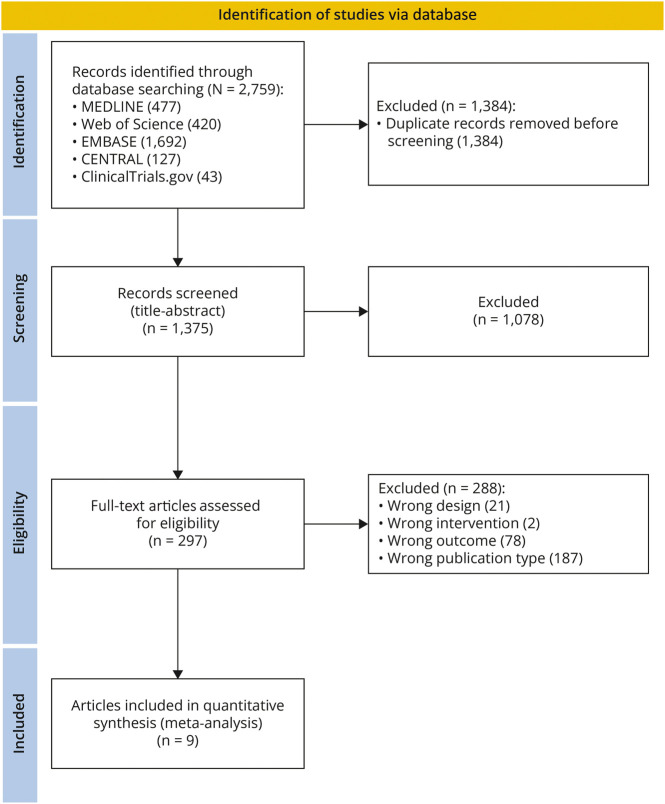
PRISMA Flowchart for Study Selection PRISMA = Preferred Reporting Items for Systematic Review and Meta-Analysis.

The initial literature search yielded 2,766 results. After the removal of 784 duplicates, 1,852 records were excluded based on title and abstract inspection. Full-text review was conducted on 69 articles, ultimately resulting in the inclusion of 9 studies in the quantitative analysis. Figure 1 shows the PRISMA flow diagram of the systematic literature search, study selection, and reasons for exclusion.

### Baseline Characteristics of the Included Studies

Nine observational studies were included,^7,9-16^ encompassing a total of 435 patients with MHVs who experienced an ICH and subsequently resumed AC (Table 1 and eTable 2). The studies were conducted over a broad time span, ranging from the late 1970s to 2022. The patient age across studies ranged from a mean of 54.1 to 75 years. The proportion of male patients varied, with some studies reporting as low as 25% and others up to 70%. Atrial fibrillation was variably reported, with some studies noting its presence in more than half of the cohort. The number of days AC was withheld ranged from a mean of 1 day to over 19 days, and the decision to restart AC was generally made within 2 weeks of the index hemorrhage in most studies. The follow-up duration varied considerably, ranging from a few days (e.g., 6 days^13^) to several months or years (e.g., 23.2 months^10^). Most studies used a mean or median follow-up period between 14 and 90 days. The 2 largestest cohorts of patients were respectively composed by 165 patients.^7^ followed by Sakusic et al.^16^ (141 patients), both of which contributed substantially to the pooled analyses.

**Table 1:** Study Characteristics

Study	Study period	Total MHV population with ICH	Duration of AC therapy	Age, years^b^	Sex, male, n (%)	AF, n (%)	Type of ICH, lobar, n (%)	Days AC discontinued	Patients with MHVs and ICH restarted on AC	Duration of follow-up
Babikian et al.^9^	Na	6	69.3 mo (mean)	66.5 (mean)	3 (50)	Na	Na	19 (mean)	6	6 mo
Butler and Tait^10^	1994–1997	16^a^	86 mo (mean)	60.2 (mean)	Na	Na	Na	7 (median)	13	23.2 mo (mean)
Leker and Abramsky^11^	1989–1996	4	9.75 y (mean)	60 (mean)	1 (25)	3 (75)	Na	1 (mean)	4	Na
Wijdicks et al.^12^	1976–1997	39	6 y (median)	69 (median)	27 (69)	12 (31)	10 (26)	8 (mean)	26	Na
Bertram et al.^13^	1992–1997	10	Na	54.1 (mean)	7 (70)	3 (30)	7 (70)	1.4 (mean)	10	6 (d)
Kuramatsu et al.^14^	2006–2010 2011–2015	137	Na	70 (mean)	90	Na	27 (19)	6.7 (mean)	66	90 (d)
Kang et al.^15^	2013–2017	6	Na	68.3 (mean)	4 (66.7)	6 (100)	4 (67.7)	8.5 (mean)	4	14.5 (d)
Barra et al.^7^	2000–2018	184	Na	65.3 (mean)	116 (63)	93 (50.8)	48 (26.1)	12.7 (mean)	165	30 (d)
Sakusic et al.^16^	2000–2022	171	Na	75 (mean)	117 (68.4)	102 (59.6)	Na	10 (median)	141	48 (d)

Abbreviations: AC = anticoagulant; AF = atrial fibrillation; ICH = intracranial hemorrhage; MHVs = mechanical heart valves.

a Includes 1 case with intraspinal hemorrhage.

^b^Calculated for the study analysis.

### Ischemic Stroke Before VKA Reinitiation After ICH

**Figure 2:**
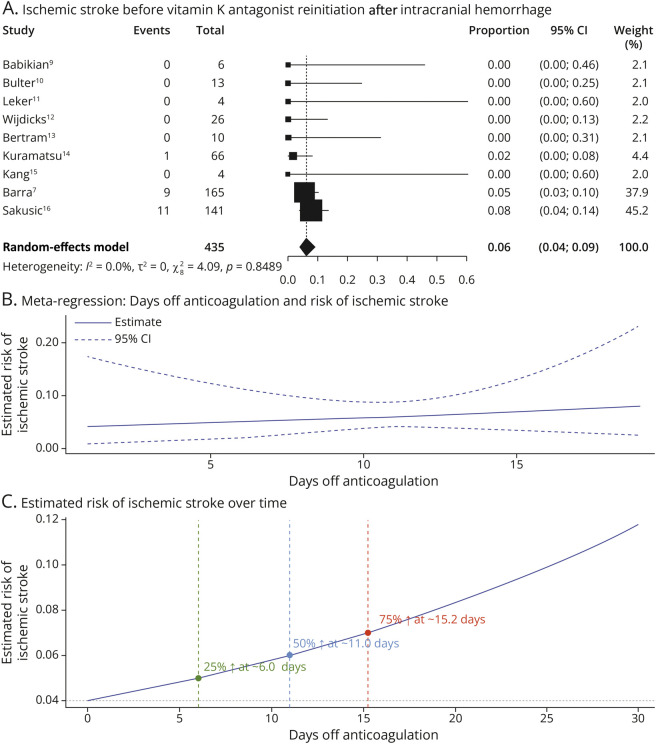
Risk of Ischemic Stroke Before VKA Reinitiation After Intracranial Hemorrhage (A) Forest plot of proportions of ischemic stroke events occurring before anticoagulant resumption across individual studies. (B) Meta-regression analysis exploring the association between the number of days off anticoagulation and the risk of ischemic stroke. The solid line represents the estimated risk, and the dashed lines represent the 95% CI. (C) Estimated cumulative risk of ischemic stroke over time off anticoagulation. The risk increases progressively with longer interruption of anticoagulation. Markers indicate the estimated number of days at which 25% (6 days), 50% (11 days), and 75% (15.2 days) of the ischemic events are expected to occur. VKA = vitamin K antagonist.

Overall, a total of 21 ischemic stroke events were observed. The pooled incidence of AIS before AC resumption was 6.1% (95% CI 4.1%–8.9%). Between-study heterogeneity was negligible (τ^2^ = 0, *I*^2^ = 0.0%, *Q* = 4.09, *p* = 0.849), indicating consistency across studies (Figure 2, panel A). Leave-one-out sensitivity analysis confirmed the stability of the pooled estimate, with proportions ranging from 4.9% (95% CI 2.9%–8.2%) to 6.4% (95% CI 3.9%–10.4%)^7^ (eFigure 1). No individual study had a disproportionate influence on the results. Visual inspection of the funnel plot showed no asymmetry, supporting the absence of evident publication bias (eFigure 2). Meta-regression revealed no significant association between the number of days off AC and the risk of AIS (coefficient: 0.0388, SE = 0.0765, *p* = 0.6123) (Figure 2, panel B). The model explained none of the variance (*R*^2^ = 0%), and residual heterogeneity remained null. Based on the meta-regression model, the estimated baseline risk of ischemic stroke before AC resumption was 4.0%. The model predicted that a 25% increase in risk (to 5.0%) would occur after approximately 6.0 days, a 50% increase (to 6.0%) after 11.0 days, and a 75% increase (to 7.0%) after 15.2 days of AC interruption. These inflection points were illustrated in a time-risk trajectory curve (Figure 2, panel C).

### Recurrence of ICH After VKA Reinitiation Following ICH

**Figure 3:**
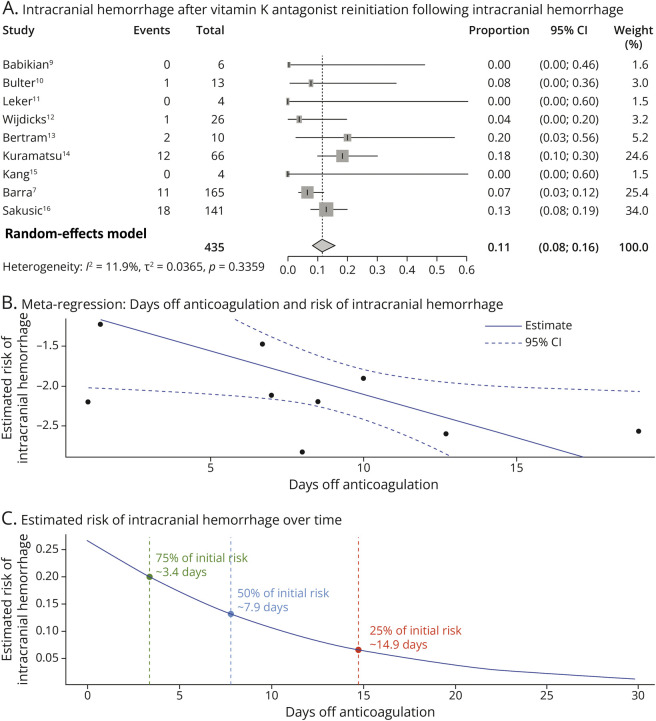
Risk of Intracranial Hemorrhage After VKA Reinitiation Following Previous Intracranial Bleeding (A) Forest plot showing the proportion of patients who experienced recurrent intracranial hemorrhage after VKA reinitiation in each study. (B) Meta-regression evaluating the association between the number of days off anticoagulation and the risk of recurrent intracranial hemorrhage. The plot indicates a decreasing risk with longer duration off anticoagulation. The solid blue line represents the estimated association, and dashed lines represent the 95% CI. (C) Estimated cumulative risk of recurrent intracranial hemorrhage over time since anticoagulation interruption. The curve shows a progressive decrease in risk. Key thresholds indicate when the risk drops to 75% (∼3.4 days), 50% (∼7.9 days), and 25% (∼14.9 days) of the initial value. VKA = vitamin K antagonist.

Overall, 45 recurrent ICH events were observed after AC resumption. The pooled incidence of ICH recurrence was 11.4% (95% CI 8.2%–15.6%), estimated using a random-effects model with logit transformation (Figure 3, panel A). Between-study heterogeneity was low, with τ^2^ of 0.0365, τ of 0.1911, and *I*^2^ of 11.9% (95% CI 0.0%–53.4%). The test for heterogeneity was not statistically significant (*Q* = 9.08, *df* = 8, *p* = 0.336), indicating that the variability across studies may be due to chance. Leave-one-out sensitivity analysis showed that the overall estimate was stable across all iterations (eFigure 3). The pooled recurrence proportion ranged from 9.9% (95% CI 7.2%–13.5%) to 13.8% (95% CI 10.1%–18.6%) No individual study disproportionately influenced the overall result, and heterogeneity remained low to moderate across all exclusions, with *I*^2^ varying between 0% and 22.8%. Visual inspection of the funnel plot showed no asymmetry (eFigure 4). These findings confirm the robustness of the pooled estimate. Meta-regression analysis revealed a significant inverse association between the number of days off AC and the risk of recurrent ICH. The regression coefficient was −0.1094 (SE = 0.0517; *p* = 0.0345), indicating that each additional day without AC was associated with a statistically significant reduction in the log-odds of recurrence. The model explained all observed heterogeneity, and the test for residual heterogeneity was nonsignificant (*p* = 0.708) (Figure 3, panel B). Using the regression model, we identified that a 25% relative reduction in risk occurred at approximately 3.4 days, a 50% reduction at 7.9 days, and a 75% reduction at 14.9 days after AC resumption. These thresholds were visualized in a risk trajectory curve generated from the model (Figure 3, panel C).

### Time-Dependent Risk Modeling of AIS and ICH After AC Interruption

**Figure 4:**
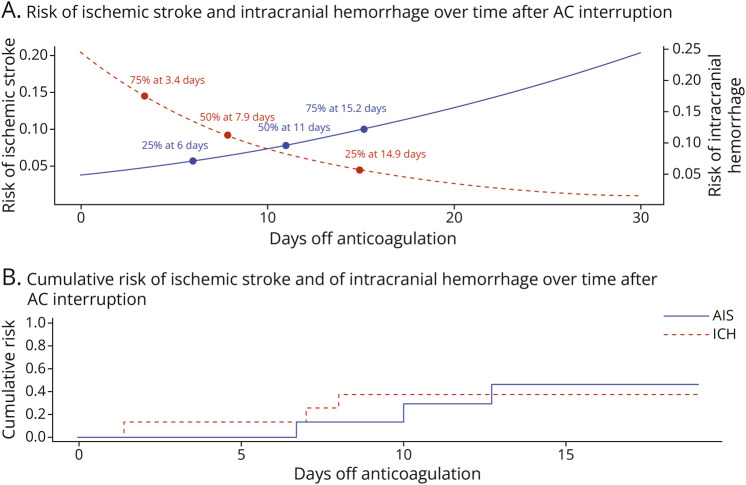
Comparative Evolution of Ischemic Stroke and Intracranial Hemorrhage Risk After AC Interruption (A) Superimposed risk curves showing the estimated risk of ischemic stroke (blue solid line) and recurrent intracranial hemorrhage (red dashed line) over time after AC interruption. Key thresholds are indicated for both outcomes at 25%, 50%, and 75% of risk variation: ischemic stroke risk increases over time while hemorrhagic risk decreases. The intersection point suggests a potential window of optimal balance for resumption. (B) Cumulative incidence curves of AIS (blue line) and ICH (red dashed line) based on days off AC. The curves depict the relative timing and cumulative risk of both events, highlighting a temporal tradeoff between ischemic and hemorrhagic complications. AC = anticoagulation; AIS = acute ischemic stroke; ICH = intracranial hemorrhage.

The modeled trajectory showed opposing trends for AIS and ICH risk over time (Figure 4, panel A). The estimated baseline risk of ICH at day 0 was 20%, which declined progressively with longer AC interruption. A 25% risk reduction in ICH occurred at approximately 6 days, 50% at 7.9 days, and 75% at 3.4 days. Conversely, the estimated baseline risk of AIS at day 0 was 4.0% and increased over time, with a 25% increase at 6.0 days, 50% at 11.0 days, and 75% at 15.2 days. This visualization highlights the dynamic interplay between hemorrhagic and thrombotic risks. A temporal “window” around 10–15 days may offer a clinically relevant balance point, where the bleeding risk has declined substantially but the thrombotic risk has remained within an acceptable range (Figure 4, panel B).

### Valve Thrombosis Before VKA Reinitiation After ICH

Nine studies reported valve thrombosis events before AC resumption, for a total of 435 patients and 9 events. The pooled incidence of valve thrombosis was 3.3% (95% CI 1.9%–5.6%), estimated using a random-effects model with logit transformation. Between-study heterogeneity was negligible, with an τ^2^ of 0 and *I*^2^ of 0.0% (95% CI 0.0%–64.8%). The test for heterogeneity was not statistically significant (*Q* = 3.58, *df* = 8, *p* = 0.893), indicating a high consistency of findings across studies (eFigure 5).

### Death After VKA Reinitiation Following ICH

The pooled incidence of mortality was 4.9% (95% CI 2.0%–11.5%), estimated using a random-effects model with logit transformation. Moderate heterogeneity was observed across studies, with an *I*^2^ of 37.4% (95% CI 0.0%–71.2%) and τ^2^ of 0.676. However, the test for heterogeneity did not reach statistical significance (*Q* = 12.78, *df* = 8, *p* = 0.120), suggesting that the variation between study results may be due to random error (eFigure 6).

### Risk of Bias

**Figure 5:**
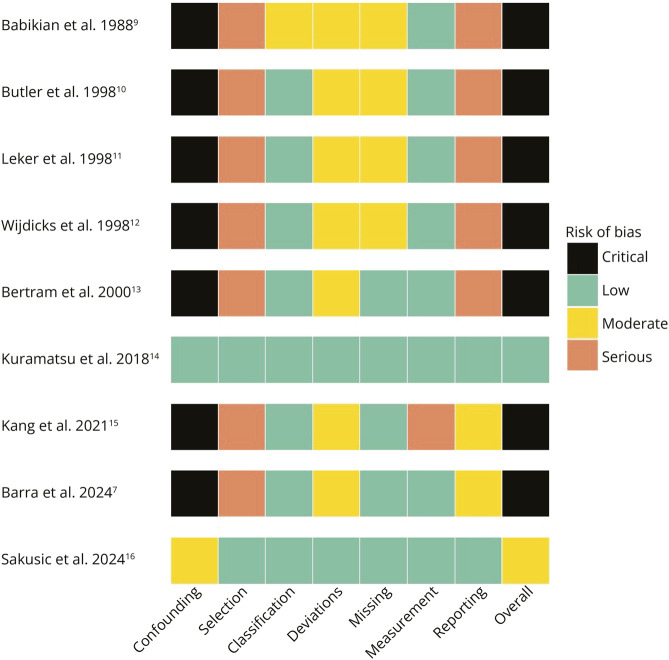
Summary of Risk-of-Bias Domains for Each Included Study, Evaluated According to the ROBINS-I Tool The 7 domains assessed are confounding, selection of participants, classification of interventions, deviations from intended interventions, missing data, measurement of outcomes, and selection of the reported result. Colors indicate the level of bias risk: black = critical, red = serious, yellow = moderate, and green = low. ROBINS-I = Risk Of Bias In Non-randomized Studies of Interventions.

According to the risk-of-bias summary (Figure 5), the study by Kuramatsu et al.^17^ was the only one rated at low risk across all ROBINS-I domains, including the overall judgment. This finding seems robust and is supported by the use of a large, prospectively collected multicenter cohort, comprehensive adjustment for confounding, and transparent outcome reporting. All other studies were judged to have a critical overall risk of bias, except for 1 study,^16^ which was rated as having a moderate overall risk, based on the adjustment for major confounders despite a limited sample size. The domain most frequently associated with high risk was bias due to confounding, rated as critical in 7 studies. This reflects the predominance of retrospective designs and uncontrolled case series, with no comparators or adjustment strategies. Bias in selection of participants was also commonly rated as serious, especially in earlier studies with unclear or selective inclusion methods. Only 2 studies^16,17^ were rated as low risk in this domain. Classification of interventions, missing data, and measurement of outcomes were generally rated at low risk, with the exception of 1 study where outcome ascertainment was not systematically described and thus judged at serious risk. Bias due to deviations from intended interventions was typically judged as moderate, reflecting real-world variation in clinical decisions without protocolization. Selective reporting bias was rated serious in older case series where outcomes were inconsistently reported and no prospective protocol was available. However, in more recent studies, the reporting domain was consistently rated as low.

Additional analyses are reported in eMethods and eFigures 7–12.

## Discussion

This systematic review and meta-analysis provides an updated and focused synthesis of current evidence regarding the timing and safety of AC reinitiation in patients with MHVs after ICH. Our findings, derived from 9 observational studies including 435 patients, provide the most comprehensive estimate to date of event rates in this high-risk population: 11.4% for recurrent ICH, 6.1% for ischemic stroke, 3.3% for valve thrombosis, and 4.9% for death. These results highlight the precarious clinical challenge of balancing the competing risks of hemorrhagic recurrence and thromboembolic complications after ICH. A key element of novelty of this analysis lies in its use of meta-regression to model the relationship between time off AC and event risk. Our results suggest that the risk of ICH recurrence decreases significantly with longer AC interruption. Specifically, a 50% reduction in risk is expected at approximately 7.9 days, with a 25% and 75% reduction at 3.4 and 14.9 days, respectively. Conversely, the thromboembolic risk, reflected by ischemic stroke occurrence, shows a slow but progressive increase over time. The model estimated a baseline stroke risk of 4.0% at day 0, with a 50% increase occurring after 11 days. These opposing temporal trajectories underscore a potential “window of opportunity” between 10 and 15 days after ICH where the balance of hemorrhagic and thrombotic risk may be most favorable.

A previous meta-analysis^18^ did not report an advanced modeling of time-dependent risk. Their pooled ICH recurrence rate was slightly higher (13%) compared with ours (11.4%). In addition, rates of thromboembolic events, including valve thrombosis (7%) and ischemic stroke (10%), were also higher than in our study. Notably, the earlier review included 23 studies with only 108 patients, most of whom were from small or anecdotal reports. By contrast, our analysis is based on larger cohorts, more rigorous inclusion criteria, and statistical methods that improve precision and generalizability. Moreover, in contrast to this previous meta-analysis,^18^ our analysis included exclusively patients who resumed AC after ICH while the previous meta-analysis included both patients who resumed and those who did not, complicating direct estimation of postresumption risks. By isolating patients who actually restarted VKAs, our findings reflect the real-world risks clinicians face when considering reinitiation of AC, thereby enhancing clinical relevance. Our findings align partially with a previous large multicenter analysis^14^ of patients with VKA-associated ICH, including a subgroup with MHVs. In their analysis, therapeutic AC resumed within 13 days was associated with increased hemorrhagic complications while thromboembolic events occurred at a lower rate in patients who remained off AC during hospitalization. However, the authors did not provide pooled estimates of risks or identify specific time thresholds, and their composite outcomes included both symptomatic and asymptomatic ICH recurrence, potentially overestimating the hemorrhagic risk. Conversely, a retrospective analysis^7^ of 184 patients with MHVs and ICH, did not show a statistically significant difference in thrombotic or hemorrhagic outcomes when AC was resumed ≤7 days vs between 7 and 30 days. However, those who did not resume AC within 30 days had significantly higher ischemic stroke and mortality rates. Their approach, however, grouped heterogeneous ICH types and applied categorical timing windows, potentially limiting temporal resolution. Our continuous meta-regression approach offers a more nuanced temporal risk trajectory, allowing for clinically interpretable benchmarks such as the “break-even” point between bleeding and ischemia.

The low heterogeneity observed across most outcomes—especially ICH recurrence and valve thrombosis—supports the reliability of our pooled estimates. The exception was mortality (*I*^2^ = 37%), possibly reflecting variability in follow-up duration, reporting standards, or underlying comorbidities. Furthermore, our leave-one-out sensitivity analysis confirmed the robustness of the pooled estimates and identified no single study as an outlier, further strengthening confidence in our conclusions.

This meta-analysis presents several strengths. First, we used a robust statistical methodology, including meta-regression and leave-one-out sensitivity analyses, which enhanced the reliability and interpretability of results. Second, the included studies represent real-world clinical practice, without excessive exclusion criteria or artificial trial settings, thereby increasing external validity. Third, the geographic diversity of the cohorts included—spanning Europe, North America, and Asia—supports generalizability across different health care contexts. Finally, we observed low statistical heterogeneity for most outcomes, further reinforcing the consistency of the findings. Our analysis has several limitations. All included studies were observational, subject to confounding, selection bias, and outcome misclassification. For instance, patients deemed at lower risk may have been selected for earlier AC reinitiation, potentially underestimating true bleeding risks. Conversely, higher risk individuals may have been excluded from resumption entirely, inflating perceived thrombotic risk if they were not captured. The retrospective nature of most studies also suggests that important clinical variables—such as ICH volume, location, or control of blood pressure—were not uniformly reported. Still, granular clinical data were often missing. Information about valve type (e.g., mitral vs aortic), AC intensity (e.g., international normalized ratio target), and use of bridging therapy (e.g., low-molecular-weight heparin) was inconsistently available. The potential influence of these variables on bleeding or thrombotic outcomes remains unquantified. Similarly, the effect of concomitant antiplatelet therapy—an important modifier of hemorrhagic risk—was rarely reported. An important limitation of this meta-analysis is the lack of granular data regarding the characteristics of the index ICH, such as hematoma volume, location, and underlying etiology. In particular, none of the included studies reported a formal diagnosis of probable cerebral amyloid angiopathy, which is now recognized as a major predictor of ICH recurrence. This absence may reflect the retrospective nature of several studies and the period during which many were conducted. Nonetheless, the variability in ICH etiology across included cohorts reinforces the real-world relevance of our findings.

In conclusion, our analysis suggests that a delay of approximately 10–15 days before resuming AC may optimize the balance between hemorrhagic and thromboembolic risk in patients with MHVs after ICH. These findings offer a data-driven framework for clinical decision making, although prospective studies with patient-level data and standardized definitions are needed to validate and refine these estimates.

## Glossary

AC: anticoagulation
AIS: acute ischemic stroke
ICH: intracranial hemorrhage
MHV: mechanical heart valve
ROBINS-I: Risk Of Bias In Non-randomized Studies of Interventions
VKA: vitamin K antagonist

## References

[1] Al-Ahmad AM, Hartnett-Daudelin D, Salem DN. Antithrombotic therapy for prosthetic valves: routine treatment and special considerations. Curr Cardiol Rep. 2001;3(1):85-89. doi:10.1007/s11886-001-0015-z 11139804

[2] Ananthasubramaniam K, Beattie JN, Rosman HS, Jayam V, Borzak S. How safely and for how long can warfarin therapy be withheld in prosthetic heart valve patients hospitalized with a major hemorrhage? Chest. 2001;119(2):478-484. doi:10.1378/chest.119.2.478 11171726

[3] Akhtar RP, Abid AR, Zafar H, Khan JS. Aniticoagulation in patients following prosthetic heart valve replacement. Ann Thorac Cardiovasc Surg. 2009;15(1):10-17. 19262444

[4] Verheugt FWA. Anticoagulation resumption after intracranial haemorrhage with mechanical valves: a data-free zone. Eur Heart J. 2018;39(19):1724-1725. doi:10.1093/eurheartj/ehy116 29538640

[5] Di Biase L. Use of direct oral anticoagulants in patients with atrial fibrillation and valvular heart lesions. J Am Heart Assoc. 2016;5(2):e002776. doi:10.1161/JAHA.115.002776 26892528 PMC4802477

[6] Dowlatshahi D, Butcher KS, Asdaghi N, et al. Poor prognosis in warfarin-associated intracranial hemorrhage despite anticoagulation reversal. Stroke. 2012;43(7):1812-1817. doi:10.1161/STROKEAHA.112.652065 22556194

[7] Barra ME, Forman R, Long-Fazio B, et al. Optimal timing for resumption of anticoagulation after intracranial hemorrhage in patients with mechanical heart valves. J Am Heart Assoc. 2024;13(10):e032094. doi:10.1161/JAHA.123.032094 38761076 PMC11179836

[8] Steiner T, Purrucker JC, Aguiar de Sousa D, et al. European Stroke Organisation (ESO) and European Association of Neurosurgical Societies (EANS) guideline on stroke due to spontaneous intracerebral haemorrhage. Eur Stroke J. 2025. doi:10.1177/23969873251340815 PMC12098356 40401775

[9] Babikian VL, Kase CS. Resumption of anticoagulation during hypertensive cerebral hemorrhage with prosthetic heart valve. Stroke. 1988;19(3):407-408. 3354031

[10] Butler AC, Tait RC. Restarting anticoagulation in prosthetic heart valve patients after intracranial haemorrhage: a 2-year follow-up. Br J Haematol. 1998;103(4):1064-1066. doi:10.1046/j.1365-2141.1998.01078.x 9886320

[11] Leker R, Abramsky O. Early anticoagulation in patients with prosthetic heart valves and intracerebral hematoma. Neurology. 1998;50(5):1489-1491. doi:10.1212/wnl.50.5.1489 9596017

[12] Wijdicks EFM, Schievink I, Brown RD, Mullany CJ. The dilemma of discontinuation of anticoagulation therapy for patients with intracranial hemorrhage and mechanical heart valves. Neurosurgery. 1998;42(4):769-773. doi:10.1097/00006123-199804000-00053 9574641

[13] Bertram M, Bonsanto M, Hacke W, Schwab S. Managing the therapeutic dilemma: patients with spontaneous intracerebral hemorrhage and urgent need for anticoagulation. J Neurol. 2000;247(3):209-214. doi:10.1007/s004150050565 10787117

[14] Kuramatsu JB, Sembill JA, Gerner ST, et al. Management of therapeutic anticoagulation in patients with intracerebral haemorrhage and mechanical heart valves. Eur Heart J. 2018;39(19):1709-1723. doi:10.1093/eurheartj/ehy056 29529259 PMC5950928

[15] Kang JH, James ML, Gibson A, et al. Anticoagulation after spontaneous intraparenchymal hemorrhage in patients with mechanical heart valves and concomitant atrial fibrillation. J Neuroanaesth Crit Care. 2021;8(3):203-207. doi:10.1055/s-0041-1735653

[16] Sakusic A, Rabinstein AA, Anisetti B, et al. Timing of anticoagulation resumption and risk of ischemic and hemorrhagic complications in patients with ICH and mechanical heart valves. Neurology. 2024;103(4):e209664. doi:10.1212/WNL.0000000000209664 39102615

[17] Kuramatsu JB, Gerner ST, Schellinger PD, et al. Anticoagulant reversal, blood pressure levels, and anticoagulant resumption in patients with anticoagulation-related intracerebral hemorrhage. JAMA. 2015;313(8):824-836. doi:10.1001/jama.2015.0846 25710659

[18] Alkherayf F, Xu Y, Gandara E, Westwick H, Moldovan ID, Wells PS. Timing of vitamin K antagonist re-initiation following intracranial hemorrhage in mechanical heart valves: systematic review and meta-analysis. Thromb Res. 2016;144:152-157. doi:10.1016/j.thromres.2016.06.014 27352237

